# Detection and quantification of pathogens in saliva of adolescents with cerebral palsy: a cross-sectional study

**DOI:** 10.3389/fdmed.2023.1208243

**Published:** 2023-12-15

**Authors:** Rosemeire Arai Yoshida, Tiago Bertola Lobato, Renata Gorjão, Lucas Santiago França, Lívia Araujo Alves, Maria Teresa Botti Rodrigues Santos

**Affiliations:** ^1^ Postgraduate Program in Dentistry, Department of Individuals with Special Needs, Cruzeiro do Sul University, São Paulo, Brazil; ^2^ Postgraduate Interdisciplinary Program of Health Sciences, Cruzeiro do Sul University, São Paulo, Brazil; ^3^ Department of Dentistry, Postgraduate Program in Dentistry, Cruzeiro do Sul University, São Paulo, Brazil

**Keywords:** cerebral palsy, periodontal disease, *Porphyromonas gingivalis*, saliva, q-PCR

## Abstract

**Background:**

Elevated levels of inflammatory mediators in saliva have been described in individuals with cerebral palsy (CP).

**Objective:**

The aim of this study was to detect and quantify the pathogens *Porphyromonas gingivalis, Aggregatibacter actinomycetemcomitans, Fusobacterium nucleatum and Prevotella intermedia* in the saliva of adolescents with CP.

**Methods:**

This is a cross-sectional study with adolescents with CP to detect periodontopathogens from saliva samples. Initially, saliva was collected from the CP (*n* = 34) and control groups (*n* = 31), followed by the gingival index (GI) for distribution of the groups of individuals with CP and control with gingivitis (bleeding on probing ≥ 10%) and without gingivitis. Bacterial DNA was extracted from saliva samples for detection of periodontopathogens by quantitative PCR (q-PCR). Data were analyzed by Mann–Whitney and Kruskal–Wallis tests, analysis of variance *t*-test (ANOVA) with Tukey–Kramer *post-hoc* tests (*p* < 0.05).

**Results:**

The quantification of DNA of periodontopathogens in saliva samples showed that adolescents with CP present a variability (22.93–39.56) in the detection of *P. gingivalis* and that some subjects with CP and gingivitis (*n* = 4) present high quantification of *P. gingivalis* (ranging 39.56–37.65), although no significant difference was found between the groups (*p* > 0.05). A significant contrast was observed for the pathogen *P. intermedia* when comparing the difference in the control group (*p* = 0.0396). No major differences were detected in the quantification of periodontopathogens evaluated between the control group and CP.

**Conclusion:**

Adolescents with CP showed variability in the detection of DNA of periodontopathogens, especially a great variation in the detection of *P* gingivalis in saliva of CP with gingivitis.

## Introduction

The imbalance between the host's immune system and the microorganisms present in the oral cavity favors the multiplication of pathogenic microorganisms and the manifestation of oral diseases (1). Periodontal disease is highly prevalent, resulting from the inflammatory pathological state initiated in response to biofilm accumulation, considered the primary etiological factor of the disease (2). It can affect the gingival tissue and the supporting structures of the dental elements (periodontal ligament, alveolar bone and cementum). Even with the restoration of gingival health after treatment of gingivitis and periodontitis, the individual is at high risk of recurrent disease, requiring periodic monitoring to control gingival inflammation as the primary prevention of periodontitis (3, 4).

Individuals with CP (cerebral palsy) have a notable pattern of gingival inflammation and high prevalence of periodontal disease (5). In a study published by our research group (6), high levels of inflammatory mediators, including interleukin IL-1β and TNF-α, were observed in the saliva of individuals with CP. Even after periodontal treatment, there was no reduction in the level of these cytokines, hence persisting the inflammatory process (6). Periodontitis is a chronic, multifactorial, immunoinflammatory disease characterized by loss of periodontal attachment (7, 8). This begins with the interaction between the pathogenic microorganisms present in the oral cavity and the host (9). Subsequently, the signaling pathway cascade is activated, enabling the production of pro-inflammatory cytokines, enzymes responsible for tissue degeneration, and an increase in the inflammatory response. In this phase, there is a change in the composition of the oral microbiome towards dysbiosis, when Gram-positive aerobic cocci are modified into Gram-negative anaerobic rods and motile spirochetes (8, 10).

Some of the most common oral pathogens in the stages preceding periodontitis are *Porphyromonas gingivalis* (*P. gingivalis*), *Aggregatibacter actinomycetemcomitans (A. actinomycetemcomitans), Fusobacterium nucleatum (F. nucleatum) and Prevotella intermedia (P. Intermedia)* (7, 11). These are Gram-negative anaerobic pathogens that have potent individual virulence factors capable of degrading host and gingival tissue proteins (9).

*P. gingivalis* is the most studied etiological agents in the pathogenesis and in the progression of periodontal inflammation and alveolar bone loss (7, 8). It is considered the key pathogen in periodontal disease with unique molecular mechanisms, which promote its survival, persistence and immune evasion within the host; it proliferates in various cells and due to its specialized virulence factors can become highly destructive (12–14).

The saliva collection method is non-invasive, inexpensive, and easily accessible. In addition, samples can be stored, making this method convenient for screening periodontal pathogens (15). Santos et al. (16) used saliva to detect inflammatory markers in the saliva of subjects with CP with and without cervical control (16)_._

Quantifying of periopathogens in saliva samples through q-PCR of adolescents with CP can contribute to enhancing the knowledge and awareness of modulating factors of periodontal disease in this population.

To date, after systematically searching the published literature and attempting different combinations of the search terms cerebral palsy, q-PCR, saliva, *Porphyromonas gingivalis, Aggregatibacter actinomycetemcomitans, Prevotella intermedia, Fusobacterium nucleatum*, and gingival inflammation, no studies were found. Thus, the aim of this study was to detect and quantify, by q-PCR evaluation, the pathogens *P. gingivalis, A. actinomycetemcomitans, P. intermedia* and *F.nucleatum*, in the saliva of adolescents with CP. The hypothesis of the study is that there is an association between a greater amount of microorganisms in the saliva of adolescents with CP and gingival inflammation.

## Material and methods

### Ethical aspects

This study was approved by the Research Ethics Committee of Universidade Cruzeiro do Sul—Plataforma Brasil, São Paulo, Brazil (CAAE: 64810517.6.0000.8084). The informed consent form (in Portuguese called the Termo de Consentimento Livre e Esclarecido or TCLE) for the participation of children and adolescents with and without CP was obtained from the caregiver or legal guardian of each participant.

### Study design

A cross-sectional study was carried out on adolescents with a medical diagnosis of spastic CP. They either attended a reference center in São Paulo, Brazil; or were clinic patients for the course Dentistry for Patients with Special Needs (campus Liberdade and São Miguel Paulista) at Universidade Cruzeiro do Sul, from August 2019 to March 2020.

### Sample size

The sample size calculation for this study was carried out by comparing the means obtained from the gingival index (GI) of the CP group (GI; mean ± SD: 13.2 ± 11.9) with the control group (GI; mean ± SD: 3.8 ± 4.7). Considering a two-tailed confidence interval (CI) of 95%, a power of 96.91% was found (www.openepi.com/Power/PowerCross.htm) (17).

### Participants

A total of 104 adolescents with CP and normoactive were invited to participate in this study, from August 2019 to March 2020.

Inclusion criteria for both groups were: aged between 11 and 18 years old, either sexes, any ethnicity and presence of 20 dental elements. The groups were not paired, as the sample was of convenience. Furthermore, the groups showed no difference in mean age between groups (Table 1). Adolescents with progressive or neurodegenerative lesions, uncooperative, who had used antibiotics in the last month, with a history of periodontal treatment for less than one month, or who presented with a chronic systemic disease that could interfere with the periodontal tissue were excluded.

**Table 1:** Distribution of adolescents according to the presence and absence of gingivitis in the groups with cerebral palsy (CP) and normoactive (GC).

Variable	Cerebral palsy (CP)		Control group (CG)		*P*-value
	With gingivitis (*n* = 19)	w/o gingivitis (*n* = 15)	With gingivitis (*n* = 5)	w/o gingivitis (*n* = 26)	
Sex *n* (%)					
Female	4 (21.1)	9 (60.0)	4 (80.0)	16 (61.5)	0.0174*
Male	15 (78,9)	6 (40.0)	1 (20.0)	10 (38.5)	
Median Age	14.2 ± 2.2	13.1 ± 2.6	13.2 ± 2.2	14.1 ± 1.7	0.9718
Clinical standard *n* (%)					
Tetraparesis	3 (15.8)	3 (20.0)	NA	NA	
Diparesis	12 (63.1)	7 (46.7)	NA	NA	0.6158
Hemiparesis	4 (21.1)	5 (33.3)	NA	NA	
Antiepileptic *n* (%)					
GABA	4 (21.1)	3 (20.0)	NA	NA	
GABA+	6 (31.6)	0 (0.0)	NA	NA	0.0471*
Sodium	3 (15.7)	2 (13.4)	NA	NA	
Not use	6 (31.6)	10 (66.6)	Not use	Not use	
Gingival index Median (±SD)	21.9 ± 9.3^A^	2.7 ± 3.5^C^	13.9 ± 2.9^B^	2.3 ± 3.0^C^	0.05*
Severity of gingivitis *n* (%)					
mild <10%	0 (0)	15 (100)	0 (0)	26 (100)	
moderate 10%–30%	16 (84)	0 (0)	5 (100)	0	
severe >30% sites	3 (16)	0 (0)	0 (0)	0	

Values represent mean ± SD or percentage (%).

* *p*-values for group comparisons were significant at 0.05; Fisher's Exact Test, ANOVA One-Way. *n* = sample size; Different superscript letters denote statistically significant differences (*p* < 0.05).

### Clinical examination

**Figure 1:**
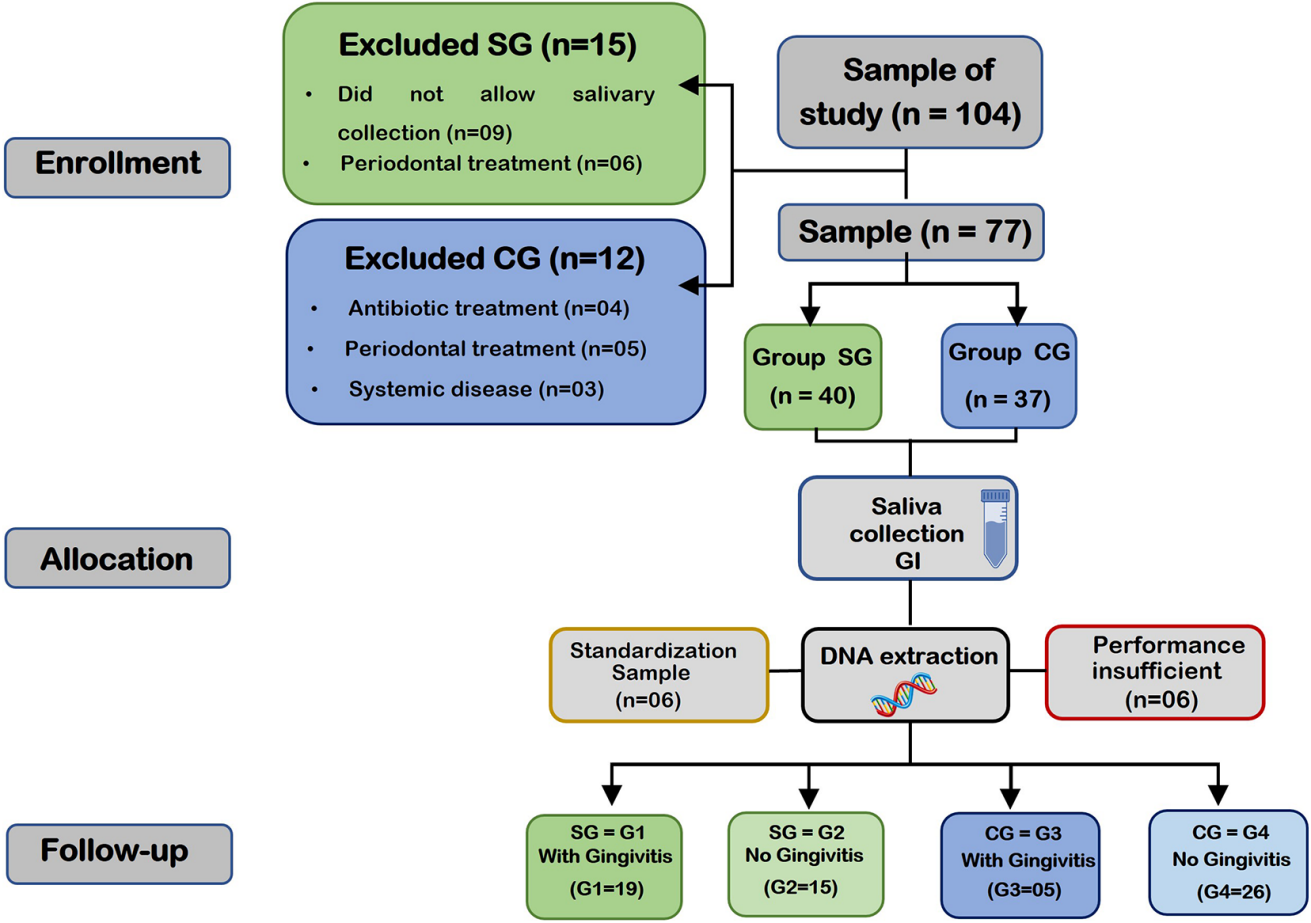
This flowchart shows the distribution of the groups: study group (SG) and control group (CG) in the enrollment, allocation and follow-up stages. The end of the flowchart shows the final number of DNA samples extracted from saliva in each group (G1, G2, G3 and G4). SG represents individuals with cerebral palsy (CP) and CG represents normoactive adolescents.

The sequence of evaluations was performed as follows: intraoral clinical examination, saliva collection and evaluation of the gingival index (GI); according to the flowchart shown (Figure 1). All clinical assessments were conducted by a single examiner (RAY) calibrated in the evaluation of the gingival index (kappa = 0.87), in a dental office with the participant properly seated in the dental chair, under the light of the reflector, obeying all biosafety norms.

#### Saliva collection

The saliva collection method used in this study was applied previously by our research group (16). The collection of saliva at rest was obtained in the morning, after a one-hour fasting period, in which the participants were asked not to eat or drink anything except water. After drying the mouth with sterile gauze, saliva was collected by means of external and spontaneous flow into a SARSTEDT®(USA) screw tube for exactly 5 min. The participants were comfortably seated in a ventilated and well-lit room, with the body leaning forward in order to facilitate the flow of saliva. The samples were transported under refrigeration to the laboratory, and kept frozen at −80°C until the moment of analysis (18, 19).

#### Gingival Index

The gingival conditions of the participants were evaluated using a millimeter periodontal probe (OMS Millimeter Probe 11.5 Single Tip—FAVA®), clinical mirror and mouth openers (if necessary). The distobuccal, buccal, mesiobuccal, mesiolingual, lingual, and distolingual sites of all teeth were probed. The gingival index was calculated by the percentage of the sum of the bleeding surfaces divided by the total number of evaluated teeth multiplied by four (20).

Individuals with gingival inflammation were considered those who presented bleeding on probing in ≥10% of the sites probed, at a probing depth of ≤3 mm in all sites evaluated, and no sites with attachment loss on probing. Individuals without gingival inflammation were considered those who presented bleeding on probing in <10% of the sites probed, at a pocket depth on probing ≤3 mm in all sites evaluated (3). No radiographic examinations were used.

### Extraction of DNA from saliva

The tubes containing the saliva samples were thawed and centrifuged at 400× *g* for 3 min at room temperature. Subsequently the sample was homogenized and an aliquot of 1.5 ml was removed for DNA extraction while, the remainder of the sample was stored in the freezer at −80°C. For the extraction, the commercial kit JetFlex™ Genomic DNA Purification Kit Invitrogen™ was used and the protocol was performed according to the manufacturer’s instructions. This aliquot was centrifuged at 12,000× *g* for 2 min at room temperature, then the pellet was resuspended in 200 µl of Phosphate-buffered saline (PBS—136.8 mM NaCl, 2.7 mM KCl, 0.9 mM NaH_2_PO_4_, pH 7.4). From this resuspended sample, an aliquot of 50 µl was removed for DNA extraction and the remainder was stored in a −20°C freezer. Cell lysis buffer (CLB) was added at a 5:1 ratio (250 µl of CLB for 50 µl of the sample), then 1 µl of proteinase K was added for each 2.5 µl of sample and incubated for 1 h at a temperature of 58°C. Afterwards, 1 µl of RNAse was added for each 5 µl of the initial sample volume and incubated again at 37°C for 5 min. Next, 150 µl of protein precipitation (PPT) buffer reagent was added, vortexed for 20 s, and centrifuged again for 10 min at 12,000× *g* at room temperature, where the pellet formed is the protein and the DNA remains in the supernatant. The DNA was purified with Isopropanol 450 µl and centrifuged for 10 min at 12,000× *g* at room temperature. Later, the sediment was washed with 1 ml of 70% ethanol -stirred using a vortex- centrifuged again for 10 min at 12,000× *g* at room temperature, and incubated at a temperature of 55°C for 10 min to evaporate all ethanol residue. The DNA pellet was resuspended in TE buffer (Tris-HCl and EDTA) 20 µl and then incubated at a temperature of 65°C for 1 h. Soon after incubation, the pellet was diluted in 30 µl of DEPC water (RNAse and DNAse free water) and analyzed in the NANODROP ONE™ (ThermoFisher Scientific, USA). It was then quantified and evaluated regarding the purity of the samples, comparing the absorbance values at 260 and 280 nm (A_260_/A_280_) and the 260/280 ratio was considered acceptable (>1.7 and <2.0) (21). While extracting DNA from the samples, six samples were discarded due to low yield, which made it impossible to extract their DNA.

### Quantitative PCR (q-PCR) amplification reaction

For the detection of pathogens in saliva, the q-PCR method was adopted using SYBR Green. Specific and universal primers were based on primers published in the literature (22, 23) and available at the GeneBank database of the National Center for Biotechnology Information (NCBI), accessed via the internet (http/www.ncbi.nlm.nih.gov/htbin-post/Entrez).

The hybridization temperature of the primers was standardized to compose the q-PCR assay template and all assays were at a temperature of 60°C. The parameters used to perform the PCR analyses were standardized. The sequence of primers used for total bacterial quantification was based on the previously designed primer (22) for highly conserved regions of bacterial 16S rRNA (P891F*—Foward:* TGGAGCATGTGGTTTAATTCGA/ P1033R*: Reverse:* TGCGGGACTTAACCCAACA). For detection of periopathogens, the following primers were used: *A. actinomycetemcomitans* (*Forward:* GAACCTTACCTACTCTTGACATCCGAA/*Reverse:*TGCAGCACCTGTCTCAAAGC) (23), *P. gingivalis* (Forward: ACATTGGGAGGGACAATGGG/*Reverse:* AGCTTCACGGAGTCGAGTTG), *F. nucleatum* (Forward: GGATTTATTGGGCGTAAAGC/Reverse: GGCATTCCTACAAATATCTACGAA) (23) and *P. intermedia* (CGGCTTTCAAGATTGGATGCTA/GTGTGAGGAAGGTGGGGATG (Supplementary Table S1).

Bacterial DNA quantification was determined using the Power SYBR™ Green PCR Master (ThermoFisher, USA) through the Quant Studio 3 equipment (ThermoFisher, Australia). Initially, 2.5 µl of the DNA sample, 0.5 µl of sense and anti-sense primers (final concentration of 200 nM) were added to 5 µl of Power SYBR™ Green PCR Master. The final volume was completed with DEPC water up to 10 µl.

The value of the relative quantification of each target gene was expressed through the comparative Ct method (Ct is the = cycle threshold, i.e., the number of cycles in which the PCR product reaches a detection threshold). Reactions are defined by the point in time (or PCR cycle) during the process where target gene amplification is detected first. This point is called the cycle threshold (Ct), where it is possible to observe the moment when the fluorescence intensity is greater than the background fluorescence. Therefore, the more expressive the amount of target DNA in the starting material and the faster there is a significant increase, a fluorescent signal will occur, producing a lower Ct (24).

All samples were processed in duplicates, for each target gene. The ratio was calculated using the Ct value of each microorganism-specific target gene, divided by the Ct value of the constitutive gene referring to the sequence for total bacteria.

With the q-PCR processing, the Melting Curve was obtained, represented by the annealing of the primers to the DNA fragments, determining and quantifying the pathogens through the change in fluorescence throughout the experiment. All the reference genes of this study were expressed at a temperature of 79°C in the Melting cycle, confirming their presence. However, for the pathogen *P. gingivalis*, observed in 36.8% of G1 and in 6.7% of G2, the amplification product was not expressive (Supplementary Figure S1). Thus, the q-PCR process was carried out again in groups G1 and G2, and the annealing of the fragments to the *P. gingivalis* primers was observed in the Melting cycle with amplification products at a temperature of 82°C.

### Statistical analysis

The participant was the unit of analysis across this study. Demographic and clinical data were computed for each participant and tabulated in tables of frequency, means and standard deviation.

The Shapiro–Wilk test was used to assume the normality of quantitative variables. Student's *t*-test (parametric data) was used for comparison between independent groups and the Wilcoxon test (non-parametric data) was used to determine significant intragroup differences. The Mann–Whitney and Kruskal–Wallis’ test (non-parametric data) was used to identify intergroup differences in relation to the microbiota between groups G1, G2, G3 and G4.

Qualitative and quantitative data from DNA extractions between groups were compared. An analysis of variance (ANOVA) was applied to compare means, with the post-hoc Tukey–Kramer test to account for multiple pairwise comparisons. To contrast the reproducibility of each method, coefficients of variation were determined to describe the percentage of variability in DNA yield relative to the mean for each DNA extraction method. Statistical analyses were performed using Prism v8, for Windows (GraphPad Software, La Jolla, CA, USA). In all tests, the significance value was set at *p* < 0.05.

## Results

Of the total of 104 adolescents invited to participate in the study, 27 participants were excluded due to uncooperative behavior (*n* = 9), previous periodontal treatment (*n* = 11), presence of chronic systemic disease (*n* = 3), use of antibiotics for less than 30 days (*n* = 4). Six participants were randomly selected for standardization of DNA extraction from pathogens. During the DNA extraction process, six samples were excluded due to insufficient yield and/or ratio <1.7.

Thus, the study group (SG) consisted of 34 adolescents with a medical diagnosis of spastic CP, divided into two groups: G1 (*n* = 19) with gingivitis and G2 (*n* = 15) without gingivitis. The control group (CG) was composed of 31 normoactive adolescents, being G3 (*n* = 5) with gingivitis and G4 (*n* = 26) without gingivitis.

The groups were homogeneous for age (*p* = 0.9718) and clinical pattern of CP (*p* = 0.6158). However, they differed in terms of sex (*p* = 0.0174) with a higher number of male participants, and gingival index (*p* < 0.05) with the CP group with gingivitis showing higher values, with 16% having severe gingivitis (Table 1).

**Figure 2:**
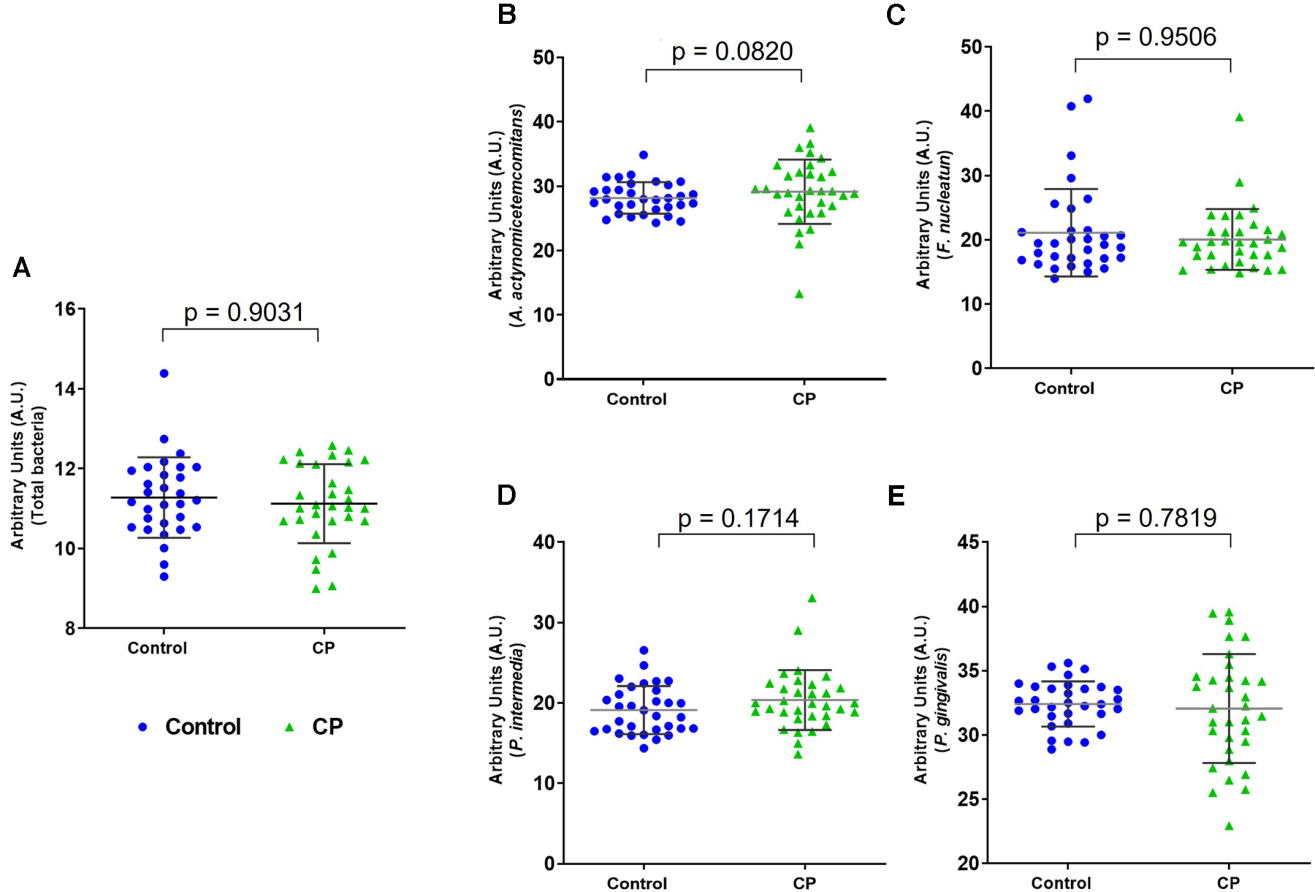
Detection of bacterial DNA by q-PCR in saliva samples from control patients and patients with cerebral palsy (CP). Graphs show the quantification of total bacterial DNA (**A**) and periodontopathogens *A. actinomycetemcomitans* (**B**), *F. nucleatum* (**C**), *P. intermedia* (**D**), and *P. gingivalis* (**E**) Relative values of mean Ct are represented in arbitrary units on a dot plot, where each point represents a sample of the group. Mann Whitney test (*p < 0.05).

Quantification of bacterial DNA by q-PCR of saliva samples from control patients and patients with CP revealed that there was no difference in the amount of total bacteria between groups (*p* > 0.05) (Figure 2A). The average detection rate of the periodontopathogen *A. actinomycetemcomitans* in the saliva of CP group was 29.18 (±3.48) while in the control group was 28.11 (±1.91), although there was no statistically significant difference (*p* = 0.0820) (Figure 2B). Interestingly, greater variability in detection of *P. gingivalis* was found in the CP group (22.93–39.56) when compared to the control group (28.87–35.59). Furthermore, the highest detection of *P. gingivalis* was found in 5 saliva samples from individuals with CP (ranging from 37.650 to 39.56) (Figure 2E).

**Figure 3:**
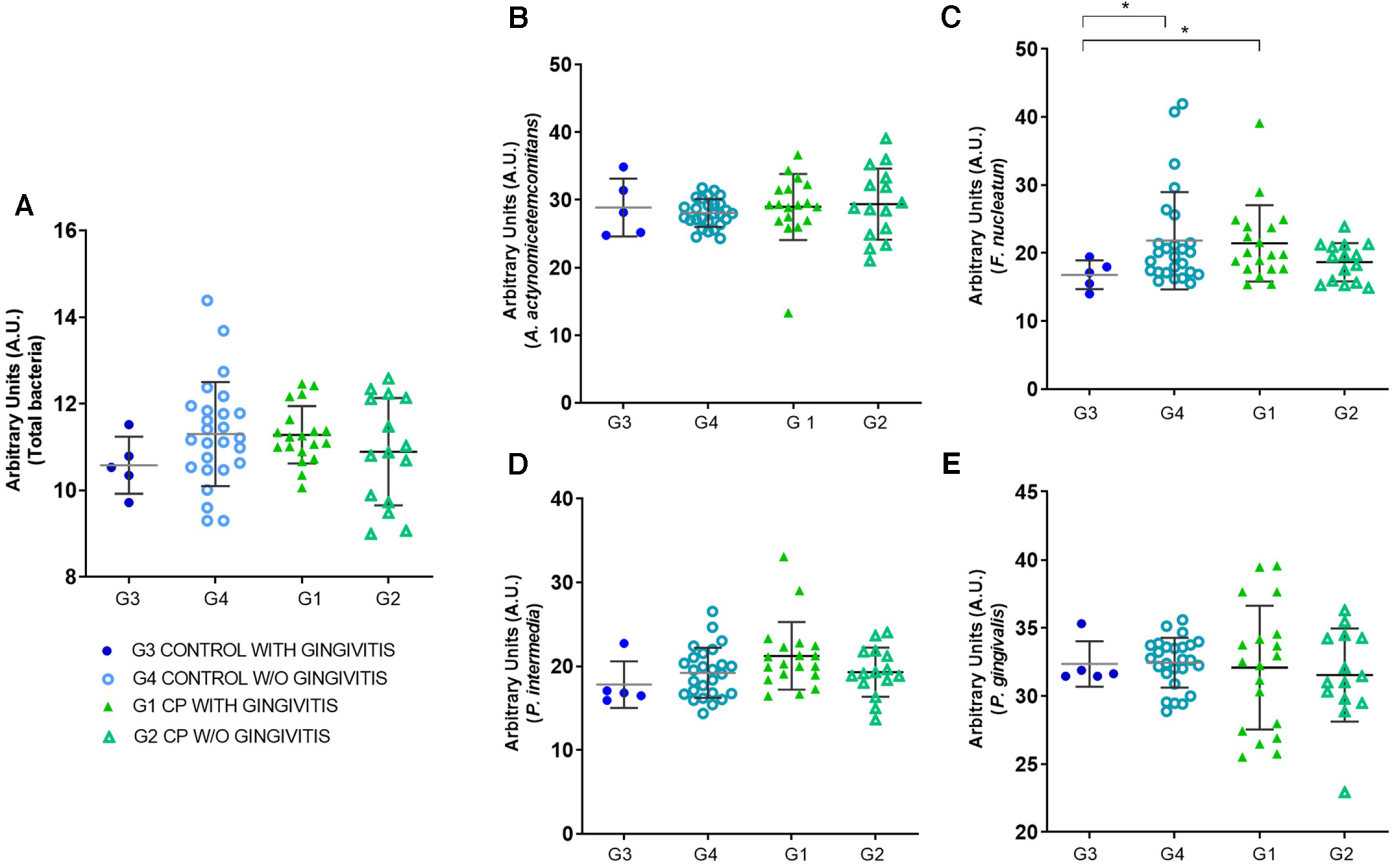
Quantification of bacterial DNA by q-PCR in groups with and without gingivitis of control (CG) and cerebral palsy (CP) subjects. Graphs show quantification of total bacterial DNA (**A**) and periodontopathogens *A. actinomycetemcomitans* (**B**), *F. nucleatum* (**C**), *P. Intermedia* (**D**), and *P. gingivalis* (**E**) in control groups with gingivitis (G3), control without gingivitis (G4), CP with gingivitis (G1) and CP without gingivitis (G2). Relative values of mean Ct are represented in arbitrary units on a dot plot, where each point represents a sample of the group. Asterisks represent the statistically significant differences (Kruskal–Wallis; *p < 0.05).

In the evaluation of the amount of DNA of periodontal pathogens by q-PCR between the groups with and without gingivitis (Figure 3), the results demonstrate that the individuals in G1 (CP with gingivitis) presented a variability (22.93–39.56) in the detection of DNA for *P. gingivalis* when compared to groups G4, G3 and G2, although no statistical difference was found between groups (Kruskal–Wallis, *p* > 0.05). It should be noted that *P gingivalis* was detected in high amounts in the saliva of 4 adolescents in the G1 group (39.47, 39.56, 37.65 and 37.65). Notably, we performed an outlier analysis, and no outlier was identified in group G1 for *P. gingivalis* detection, showing that this group has great variability, representing a characteristic of this group.

Furthermore, a greater amount of *F. nucleatum* was detected in the CP group with gingivitis when compared to the control group with gingivitis (*p* < 0.05). Curiously, the control group without gingivitis showed an increase in *F. nucleatum* when compared to the control group with gingivitis (*p* < 0.05). In the other groups, no significant differences were observed (*p* > 0.05). The dot plot graph shows (Figure 3) that the G1 group presents a greater dispersion of the samples, compatible with the higher values of gingival inflammation, especially in the G1 group for detection of *P. gingivalis*.

The diagnostic reference standard revealed that q-PCR was able to detect the target bacterial species in saliva samples. It also showed similar prevalence and bacterial counts between groups G1, G2, G3, and G4 for *A. actinomycetemcomitans* and *P. intermedia*, with no statistically significant differences. Analyzing the dot plot graphs in Figure 3, where each point represents a sample of the group, Figure 3E then exhibits a greater diversity among the samples of the *P. gingivalis* groups, showing how this periodontopathogen influences the differentiation between the groups with and without gingivitis.

Comparing the SG or CP and CG groups with respect to the ratio (obtained by the average of the Ct of the pathogen with the Ct of the gene for total bacteria), no significant difference was found in relation to the studied pathogens (*p* > 0.05). In calculating the difference (obtained by subtracting the mean Ct of the pathogen gene from the Ct of the gene for total bacteria), a significant difference was observed (*p* = 0.03158), with a greater amount of DNA for the pathogen *P. gingivalis* (Supplementary Table S2).

A significant contrast was observed for the pathogen *P. intermedia* when comparing the difference in the control group (*p* = 0.0396) in relation to the other pathogens evaluated (Supplementary Table S3).

## Discussion

This study demonstrated that adolescents with CP and gingivitis have a different amount of DNA fragments of the pathogen *P. gingivalis*. So far, this is the first study that quantified periodontal pathogens by the q-PCR method in the saliva of adolescents with spastic-type CP, which is the most prevalent and frequent type (75% of cases) among subjects with CP (25).

It is known that biofilm is the primary etiological factor of periodontal disease (2). In this, it must be considered that maintaining satisfactory oral hygiene is a daily challenge for caregivers of individuals with CP, favoring the development of periodontal disease in these individuals (26, 27). Other factors contribute to the progression of periodontal disease, such as spasticity in the masticatory muscles, reduction in the amplitude of mouth opening (28), the presence of primitive oral reflexes such as tonic bite and vomiting (29), reduced salivary flow, and the presence of intestinal constipation in subjects with CP using antiepileptic drugs (30, 31).

The use of GABA drugs and associations with it in the control of epilepsy lead to a reduction in salivary flow, favoring the development of periodontal disease. Antiepileptic drugs may cause several side effects, including gastrointestinal complications, oral dysbiosis, gingival bleeding, and increased systemic inflammation. In order to analyze whether there is a change in the oral microbiota among adolescents who use antiepileptic drugs, we compared the abundance of bacteria (total bacteria) between groups of CP users and non-users of antiepileptic drugs and found a significant positive correlation (Pearson r: 0.3449; *p* = 0.0494). The highest value of the gingival index observed in our study group with gingivitis reflects the side effects of the use of antiepileptic drugs, corroborating the results found by Ferreira et al. (32). The adolescents in the CP group with gingivitis in this study used polytherapy and had the highest gingival index values. We analyzed the bleeding index values and the use of antiepileptic medication among PCs with gingivitis (*n* = 19) and no significant positive correlation was observed (Pearson r: 0.2021; *p* < 0.4068). Hence, the use of antiepileptic medication in association should be considered as a modifier of gingival health for this population.

In the present study, it was observed that *P. gingivalis* was detected differently in samples from patients with CP and gingivitis, with greater variation in detection between saliva samples from group G1 (25.52–39.56). This analysis demonstrated variations in melting cycle temperature which was a unique finding for these subjects. The possible cause of this detection must be correlated with a characteristic of this pathogen that modulates the entire oral ecosystem through the engineering of its environment, modifying the host's immune response, altering the inflammation signaling pathways, complement system, cell cycle and apoptosis (33). *P. gingivalis* has been detected at different temperatures, which suggests that the G1 group may present a genetic variation of the pathogen. This genetic variation could justify the chronic gingival inflammatory process in adolescents. Additionally, this inflammation may evolve into periodontal disease in the future. We also observed a prevalence of the pathogen *A. actinomycetemcomitans* in the CP group in relation to the control group. Due to the dysbiosis caused in the inflamed site, the virulence factors of *A. actinomycetemcomitans* can induce an immunological paralysis where the immune response is stimulated by molecular patterns associated with other periodontopathogens (34).

One study showed the interaction and participation of *A. actinomycetemcomitans* in the activation of receptors that detect intracellular microorganisms and their products (34). This process results in the activation of multiprotein cytoplasmic complexes called inflammasomes, which activate a cell function that causes the release of interleukins inducing apoptosis (34).

Its participation along with the pathogen *P.gingivalis* makes the inflammatory process more aggressive. Furthermore, the study showed that the presence of *P.gingivalis* interacting with gingival epithelial cells and macrophages induces the production of IL-1β (33). This may explain the greater variability of this pathogen in the results presented and the greater presence of this interleukin in the saliva of patients with periodontal disease, as previously demonstrated by studies published by our group (6). Possibly, alteration in immune system components and interleukin detection in CP patients with gingivitis may explain the high variability of *P. gingivalis* detection in this group.

Another pathogen that is implicated in the inflammatory process is *P. intermedia*. It was observed a significant difference (*p* = 0.0396) in the comparison between the CP and control groups when calculating the difference value between groups (Supplementary Table S3). This pathogen has virulence factors that cause it to adhere to biofilm formation and certain strains produce twice as much biofilm as monospecies, which can result in high-level resistance to antibiotics (35), justifying the recurrence of gingival inflammation.

An interesting fact pointed out by the current study was the higher quantification of *F. nucleatum* DNA in both saliva samples from control patients without gingivitis and from CP patients with gingivitis. This finding can be explained by the dual role of *F. nucleatum* both as a commensal bacterium, being classified as a species that weakly induces defensins (34), as well as its role in the immunopathogenesis of periodontal disease, inducing the regulation of the cytokine IL-1β by epithelial cells (36). Increased IL-1β in the saliva of patients with periodontal disease was also observed in previous studies by our group (6).

Regarding the limitation of our study, a smaller number of participants was observed in the G3 group (*n* = 5) (control with gingivitis) due to the lower adherence of adolescents in this group. However, it had a similar allocation between the control groups (*n* = 31) and CP group (*n* = 34). Another limitation of our study refers to the non-performance of the plaque index, due to the difficulty in removing the evidence in the CP group, especially with the quadriplegic clinical pattern.

Even though there is a growing production of studies that address the need for oral health care and orientation for special needs patients, such production is still insufficient (37). There is a knowledge gap on the perceptions of parents and caregivers regarding the oral health care that should be offered to children and adolescents with CP, in the different levels of health care that provide services to this demographic (37).

## Conclusion

Adolescents with CP showed a variability in the detection of periodontopathogens according to bacterial DNA quantification by q-PCR. A variability in detection of *P. gingivalis* was found in the saliva of CP with gingivitis, with some adolescents having high quantification of *P. gingivalis.* This finding may be related to alterations in the immune response observed in a previous study in this group. However, more research is needed to confirm these results.

## Data Availability

The raw data supporting the conclusions of this article will be made available by the authors, without undue reservation.
