# Supplementary material for: Detection and quantification of pathogens in saliva of adolescents with cerebral palsy: a cross-sectional study
Source: Front Dent Med. 2023 Dec 15;4:1208243. doi: 10.3389/fdmed.2023.1208243 (PMC11811777; doi:10.3389/fdmed.2023.1208243)
Supplement: Supplementary file 1 [file Datasheet1.pdf]

Supplementary Material

Detection and quantification of pathogens in saliva of adolescents with cerebral palsy: a cross sectional study

Rosemeire Arai Yoshida[1], Tiago Bertola Lobato[2], Renata Gorjão[2], Lucas Santiago França[3], Lívia Araujo Alves[3], Maria Teresa Rodrigues Botti Santos[1]*

*** Correspondence:**

Profa. Dra. Maria Teresa Botti Rodrigues Santos

drsantosmt@yahoo.com.br

Supplementary Table 1. Sequence of primers used in the study.

Gene	Primer Sense	Primer Antisense
Aggregatibacter actinomycetemcomitans	GAACCTTACCTACTCTTGACATCCGAA	TGCAGCACCTGTCTCAAAGC
Porphyromonas gingivalis	ACATTGGGAGGGACAATGGG	AGCTTCACGGAGTCGAGTTG
Fusobacterium nucleatum	GGATTTATTGGGCGTAAAGC	GGCATTCCTACAAATATCTACGAA
Prevotella intermedia	CGGCTTTCAAGATTGGATGCTA	GTGTGAGGAAGGTGGGGATG
Total bacteria (16s RNA)	TGGAGCATGTGGTTTAATTCGA	TGCGGGACTTAACCCAACA

Supplementary Table 2. Comparison of the Ratio and Difference between the CP and CG groups, referring to the microorganisms studied.

RATIO			
**Microorganisms**	**CP**	**CG**	**p-valor**
P. gingivalis	3,069±0,1893	2,892 ± 0,2308	0,3278[$]
A. actynomicetemcomitans	2,595±0,076	2,517± 0,049	0,4033[$]
F. nucleatun	1,806±0,3734	1,88±0,6118	0,7718[Ŧ]
P. intermedia	1,839±0,3303	1,695±0,2086	0,4752[Ŧ]

DIFFERENCE			
**Microorganisms**	**CP**	**CG**	**p-valor**
P. gingivalis	22,86 ± 2,386	21,13±1,574	0,03158[$]
A. actynomicetemcomitans	17,62±0,834	16,91±0,435	0,4619[$]
F. nucleatun	8,96±0,7722	9,833±1,192	0,8604[Ŧ]
P. intermedia	9,257±3,57	7,832±2,484	0,4929[Ŧ]

[$] t-Student Test; [Ŧ]Mann-Whitney; Cerebral Palsy (CP); Control group (CG); *p<0,05

Supplementary Table 3. Comparison of the Difference between the CP and control groups (CG) for the evaluated pathogens.

**Variable**	**CP**	**CG**	**p-valor**
P. gingivalis	20,95 ± 3,95	21,13 ± 1,59	0,2517
A. actnomycetemcomitans	17,65 ± 4,72	16,84 ± 2,47	0,0800
F. nucleatun	9,07 ± 4,41	8,70 ± 6,33	0,1827
P. intermedia	9,32 ± 3,53	7,72 ± 2,44	0,0396*

Data comparison Shapiro-Wilk test for normality and paired and unpaired Student t-test, *p < 0.05

A B

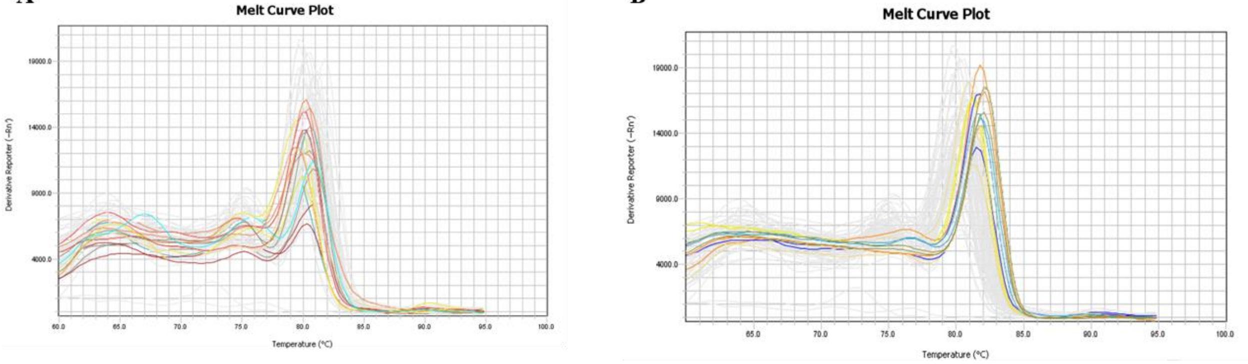

Supplementary Figure 2. Melt Curve of the pathogen *Porphyromonas gingivalis.* A) Temperature at 79°C. B) Temperature at 82°C.
